# The Genome of *Nosema* sp. Isolate YNPr: A Comparative Analysis of Genome Evolution within the *Nosema/Vairimorpha* Clade

**DOI:** 10.1371/journal.pone.0162336

**Published:** 2016-09-06

**Authors:** Jinshan Xu, Qiang He, Zhenggang Ma, Tian Li, Xiaoyan Zhang, Bettina A. Debrunner-Vossbrinck, Zeyang Zhou, Charles R. Vossbrinck

**Affiliations:** 1 College of Life Sciences, Chongqing Normal University, Chongqing, China; 2 State Key Laboratory of Silkworm Genome Biology, Southwest University, Chongqing, China; 3 Department of Math/Science, Gateway Community College, 20 Church Street, New Haven, CT 06510, United States of America; 4 Department of Environmental Science, Connecticut Agricultural Experiment Station, 123 Huntington Street, New Haven, CT 06511, United States of America; University of Lausanne, SWITZERLAND

## Abstract

The microsporidian parasite designated here as *Nosema* sp. Isolate YNPr was isolated from the cabbage butterfly *Pieris rapae* collected in Honghe Prefecture, Yunnan Province, China. The genome was sequenced by Illumina sequencing and compared to those of two related members of the *Nosema/Vairimorpha* clade, *Nosema ceranae* and *Nosema apis*. Based upon assembly statistics, the *Nosema* sp. YNPr genome is 3.36 x 10^6^bp with a G+C content of 23.18% and 2,075 protein coding sequences. An “ACCCTT” motif is present approximately 50-bp upstream of the start codon, as reported from other members of the clade and from *Encephalitozoon cuniculi*, a sister taxon. Comparative small subunit ribosomal DNA (SSU rDNA) analysis as well as genome-wide phylogenetic analysis confirms a closer relationship between *N*. *ceranae* and *Nosema* sp. YNPr than between the two honeybee parasites *N*. *ceranae* and *N*. *apis*. The more closely related *N*. *ceranae* and *Nosema* sp. YNPr show similarities in a number of structural characteristics such as gene synteny, gene length, gene number, transposon composition and gene reduction. Based on transposable element content of the assemblies, the transposon content of *Nosema* sp. YNPr is 4.8%, that of *N*. *ceranae* is 3.7%, and that of *N*. *apis* is 2.5%, with large differences in the types of transposons present among these 3 species. Gene function annotation indicates that the number of genes participating in most metabolic activities is similar in all three species. However, the number of genes in the transcription, general function, and cysteine protease categories is greater in *N*. *apis* than in the other two species. Our studies further characterize the evolution of the *Nosema/Vairimorpha* clade of microsporidia. These organisms maintain variable but very reduced genomes. We are interested in understanding the effects of genetic drift versus natural selection on genome size in the microsporidia and in developing a testable hypothesis for further studies on the genomic ecology of this group.

## Introduction

Microsporidia are unicellular eukaryotes that are obligate intracellular parasites, entering host cells by injection of the sporoplasm through a polar filament [1,2,3]. Microsporidial infections in insects are thought to be responsible for naturally occurring low to moderate insect mortality and have promise for biological control [4,5,6]. At present, more than 1,500 microsporidial species belonging to 187 genera have been reported [7,8]. The greatest numbers of species have been described from insects and fish, but microsporidia appear to infect all animal species which have been carefully examined. Microsporidia cause economic damage in sericulture [9,10], apiculture [11,12,13] and aquaculture [14,15], and as opportunistic infections are an important consideration in the AIDS epidemic as numerous microsporidial infections have been reported from immunocompromised humans [16,17]. Some groups of microsporidia, such as the Amblyosporidae, have complex life cycles [18,19] and appear to be relatively specific regarding their definitive host [20,21].

*Nosema* sp. YNPr is considered to be a parasite primarily of the "cabbage butterfly" (*Pieris rapae*), an economic pest of cruciferous crops, such as cabbage, rape, cauliflower and broccoli [4,22]. In addition, there are a number of other microsporidian species from the *Nosema/Vairimorpha* clade that have been isolated from *P*. *rapae* [23,24,25,26].

Based upon comparative small subunit ribosomal DNA (SSU rDNA) and large subunit ribosomal DNA (LSU rDNA) analyses, the genera *Nosema* and *Vairimorpha* are paraphyletic taxa clustering together in what is now referred to as the *Nosema/Vairimorpha* clade [27,28]. There are over 100 reported species from this clade and they appear to be generalist parasites with simple single-host life cycles, able to switch hosts through cross-infection and adaptation [28,29]. It has been known for some time that, in addition to the domesticated silkworm *Bombyx mori*, *Nosema bombycis* can also infect various other lepidopteran insects [29,30,31]. In fact, it appears that *N*. *bombycis*, the first described microsporidian species, isolated from *B*. *mori* [32], was an opportunistic infection originating from other Lepidoptera living in or near the mulberry fields. Host-range virulence feeding studies revealed that many of the members of the *Nosema/Vairimorpha* clade seem to be switching hosts relatively rapidly with varying degrees of infection in various tissues making such host range studies an important part of microsporidian ecology [6,33,34].

The genomes of some microsporidia are extremely compact. The genomes of *Encephalitozoon* species (a sister taxon to the *Nosema/Vairimorpha* clade) range in size from 2.3 to 2.9 x 10^6^ bp. The genomes of *Encephalitozoon* species encode roughly 2,000 genes with few introns, very short intergenic regions, and no transposable elements [35,36,37,38]. Phylogenetic analysis of the microsporidia shows that microsporidial genomes expand and contract over relatively short evolutionary time [39]. The highly compact nature of the microsporidial genome has been discussed in terms of minimal genes necessary for the survival of an obligate intracellular parasite and the origins of the microsporidia [40,37]. The reduced genome of *Enterocytozoon bieneusi* lacks some of the genes for core carbon metabolism [41] and contains highly compacted, overlapping genes [42]. The microsporidia are considered to have rapidly evolving genes while maintaining a high degree of genome synteny. The "shrinking" of the genome has been attributed to the obligate intracellular life style which "has permitted the loss of many genes whose functions can be provided by the host cell" and to the loss of introns[43]. Organism complexity is not thought to be related to genome size but perhaps to other factors such as metabolic rate, body size, population size, cell size and nucleus size [44].

The idea that genome size is a limiting factor in the rate of reproduction in prokaryotes is an attractive one, however an analysis of 214 species of bacteria and archaea suggests that in prokaryotes growth optimization is correlated with traits such as codon usage biases and shows no correlation between genome size and reproductive rate. It has been suggested that generation times may be determined by factors such as environment stability and nutrient availability [45].

Streamlining, the reduction of genome size, has been examined in a number of prokaryotic species. For most species that have been studied, streamlining appears to occur through genetic drift [46]. In thermophilic bacteria, however, it has been shown that the proportion of genomic DNA in intergenic regions decreases with smaller genome size, that there is a correlation between genome size and generation time and that the genome-wide selective constraints (dN/dS measurements) do not decrease with smaller genomes, suggesting that in thermophiles genome reduction is due to natural selection [46]. It has been suggested that cell size, which correlates with genome size, may be the direct target of natural selection; large cells may suffer fitness costs at higher temperatures [46]. Examination of bacterial genome size reduction through experimental selection suggests that genome reduction can occur over a short evolutionary time [47].

Because of their speciose nature and their presence in a wide range of insects, members of the *Nosema/Vairimorpha* clade present a good system in which to study ecological genomics of intracellular parasites. In this study we compare the genome of *Nosema*. *sp*. isolate YNPr from *P*. *rapae* collected in Honghe Prefecture, Yunnan Province, China with the genomes of two microsporidial honeybee parasites, *Nosema ceranae* and *Nosema apis*. We hypothesize that genome size is a limiting factor in the rate of reproduction of the Microsporidia and that there is an evolutionary tradeoff between having a small genome and reproducing rapidly versus having a larger genome and giving the parasite more genetic options with which to challenge a host.

## Materials and Methods

### Isolation of spores and genomic DNA extraction

**Fig 1:**
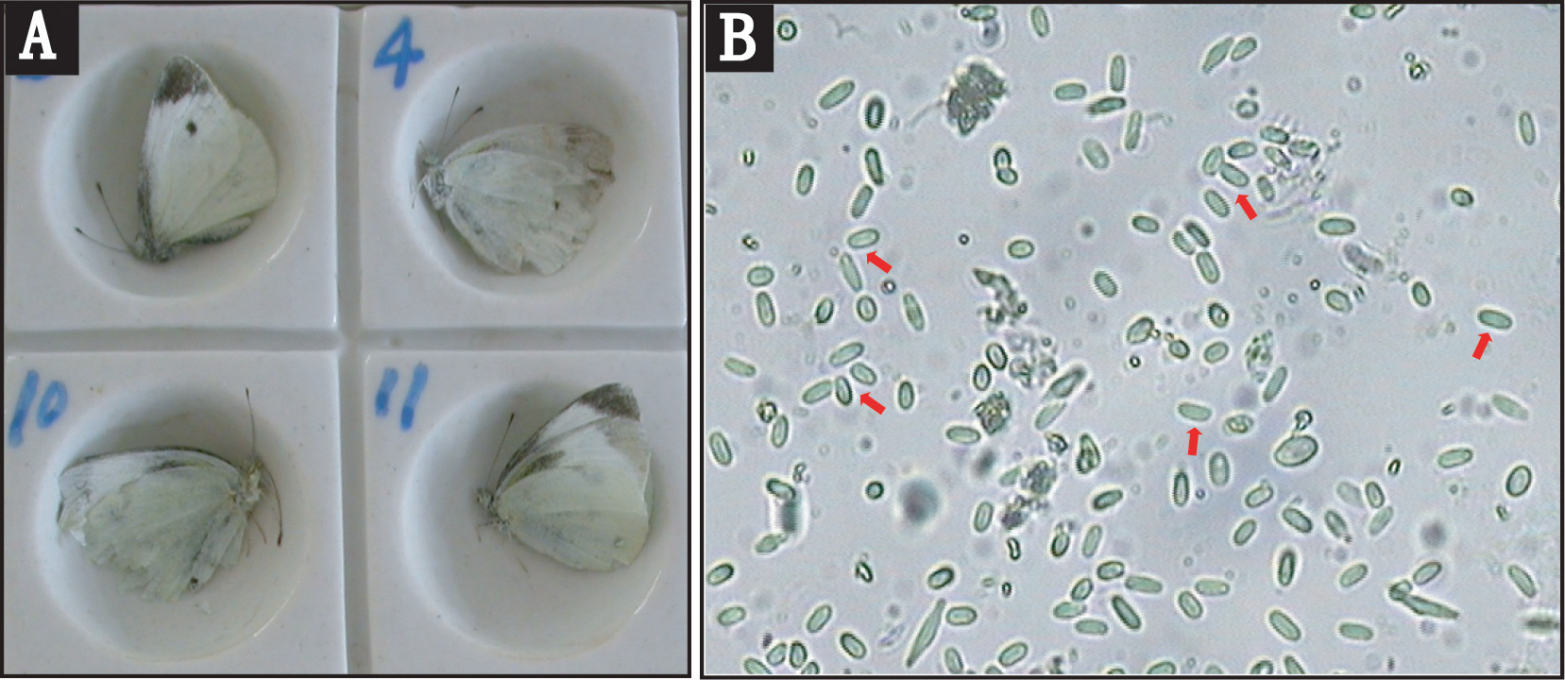
Images of host and parasite. A. Micrograph of the adult of *Pieris rapae* Linne. B. Spores of *Nosema* sp. YNPr at 400X.

Wild-caught cabbage butterflies (*P*. *rapae*) infected with microsporidia were field collected in Honghe Prefecture, Yunnan Province, China (Fig 1A), and no specific permissions were required for these locations. The wings were removed and the infected *P*. *rapae* were then disrupted with a glass homogenizer. The homogenates were filtered through three layers of cheesecloth and centrifuged at 10,000 x g for 30 seconds. The pellets were re-suspended in PBS buffer and purified by centrifugation at 1,500 x g for 30 min on a Percoll gradient. The spore band was collected and washed several times with sterile water and checked for purity with a phase contrast microscope (Fig 1B).

Approximately 1×10^9^ spores of *Nosema* sp. YNPr were extracted directly using the cetyl trimethylammonium bromide (CTAB) method [48]. The concentration and purity of the DNA were determined spectrophotometrically based on absorption readings and ratios at 260 nm and 280 nm by using a NanoDrop spectrophotometerND-1000

### Ribosomal DNA sequencing

PCR amplification was performed under the following conditions:

Samples were heated to 94°C for 5 min to denature the DNA followed by 30 cycles of: 1 min of denaturation at 94°C, 30 s of annealing at 55–58°C, and 2 min of extension at 72°C. A final extension step was carried out at 72°C for 10 min. Primers used are as follows:

SSU rRNA:     5'-CACCAGGTTGATTCTGCC-3'

                       5'-TTATGATCCTGCTAATGGTTC-3'

ITS:                 5'-TGAATGTGTCCCTGTTCTTTG-3'

                       5'-GTTAGTTTCTTTTCCTCC-3'

PCR products were run on agarose gels, excised and purified using a gel extraction kit (E.Z.N.A. ® Gel Extraction Kit) following the manufacturer’s instructions. The purified PCR products were then cloned into a pMD19-T vector using a TA Cloning Kit (TaKaRa Biotechnology, Dalian, China) and bacterial colonies were sent to Beijing Genomics Institute (BGI) Shenzhen, China for Sanger sequencing.

### Comparative rDNA analysis

The SSU rDNA and ITS rDNA sequences from *Nosema* sp. YNPr were compared to those downloaded from GenBank. Sequence alignments were performed by MUSCLE [49].Phylogenetic trees were constructed using neighbor-joining (NJ) analysis with MEGA version 5 [50]. The Maximum Composite Likelihood model was used and bootstrap support was evaluated based on 1,000 replicates.

### Genome sequencing and assembly

A genomic DNA library was prepared for Illumina HiSeq2500sequencing following the manufacturer’s instructions (Illumina). The genomic DNA was fragmented by nebulization with compressed nitrogen gas at 32psi for 9 minutes. Any overhangs were converted to blunt ends using T4 DNA polymerase and Klenow polymerase, after which an adenine nucleotide was added to the ends of double-stranded DNA using Klenow Exo-Minus polymerase (Qiagen). DNA adaptors with a single “T” base overhang at the 3’ end were ligated to the above products. The resulting DNA was then separated on a 2% agarose gel and fragments of approximately 800 bp were excised from the gel and purified (Qiagen Gel Extraction Kit). The adapter-modified DNA fragments were amplified using Illumina primers 1.1 and 2.1. The concentration of the DNA library was determined by measuring the absorbance at 260nm and the DNA was then sequenced (BGI). The paired-end reads were processed by removing adaptor and low quality (Q<30) sequences. As a result, 3,154,248 high-quality reads were obtained for a total of 1.57 million base pairs. Each read was about 100 bp in length. The paired-end reads were assembled de novo using the Velvet1.2.10 assembly program [51], with the standard option as following: insert size of 800 ± 160 bp and a Hash length of 31. The resulting scaffolds were further processed with GapFiller1.11[52] using the default settings.

### Gene prediction and annotation

The *Nosema* sp. YNPr protein-coding genes were annotated with Glimmer 3.0 software using the lower eukaryote settings [53,54]. We identified 2075 ORFs. To avoid the over-prediction of small genes, the ORFs were re-annotated by searching for transcriptional signals (CCC or GGG-like motifs) within 50 nucleotides upstream of the first or successive downstream AUG codons within the ORF [48,55]. We identified 1425 CDS by this method. The remaining 650 ORFs were further examined by searching for AT-rich regions (AT content > = 80%) within 50 nucleotides upstream of the first or successive AUG codons [55] within the ORFs. We identified a further 275 CDS using this method.

The MEME 3.0 software program was used for motif exhibition of putative transcription regulators by searching for over-represented motifs in the 50-bp upstream region of the start codon of protein-coding genes. Gene annotation was accomplished utilizing NCBI BLASTP against the GenBank non-redundant database (nr), Swiss-Prot database with a cut-off e-value of 1e-6. Gene ontology classification of the three *Nosema* species was performed using InterProScan5 software and then visualized through on-line WEGO tools (http://wego.genomics.org.cn/cgi-bin/wego/index.pl).

Comparison of genes based upon function among *N*. *ceranae*, *N*. *apis* and *Nosema* sp. YNPr was accomplished using the Clusters of Orthologous Groups (COG) protein database (http://www.ncbi.nlm.nih.gov/COG/). BLAST searches against the local COG database were performed with a cut-off e-value of 1e-6, and the best hit which contained the protein identity was used to assign the functional categories of COG based on the list of COG annotations. Similarly, proteases were identified using a BLAST search of all predicted genes against the MEROPS database (http://merops.sanger.ac.uk/cgi-bin/batch_blast) [56]. Genes with e-values less than 1e-6 were further verified as proteases by searching against the Genbank database. The best hits in the MEROPS database were used to assign the protease classes. BLASTCLUST 2.2 was used to identify homologous genes among *Nosema* sp. YNPr and three other species (*N*. *ceranae*, *N*. *apis* and *E*. *cuniculi*) with 30% identity and 50% coverage. Differences in gene length among homologous genes and the number of unique genes in each of the species were calculated using custom PERL scripts written in our lab.

The final genome and predicted protein-coding genes for *Nosema* sp. YNPr are deposited in NCBI (Project PRJNA325422) and the Silkworm Pathogen Database (SilkPathDB, http://silkpathdb.swu.edu.cn/). The Illumina data generated for *Nosema* sp. YNPr is available in the NCBI SRA (accession number SRR3673305).

### Transposon and signal peptide analysis

The assembled genomes of *Nosema* sp. YNPr, *N*. *ceranae*, *N*. *apis* and *N*. *bombycis* were checked for interspersed repeats and low complexity DNA sequences using RepeatMasker 4.05(http://www.repeatmasker.org)[57]. The transposons were identified and their lengths were calculated. The conserved reverse transcriptase domain sequences (RVT) from LTR retrotransposons were obtained through the NCBI Conserved Domains search site (http://www.ncbi.nlm.nih.gov/Structure/cdd/wrpsb.cgi) to predict the conserved domains.

Signal peptide sequences of genes for each microsporidia species were predicted by SignalP 4.1 under the default D-cutoff values (http://www.cbs.dtu.dk/services/SignalP/).

## Results

### Phylogenetic analysis

**Fig 2:**
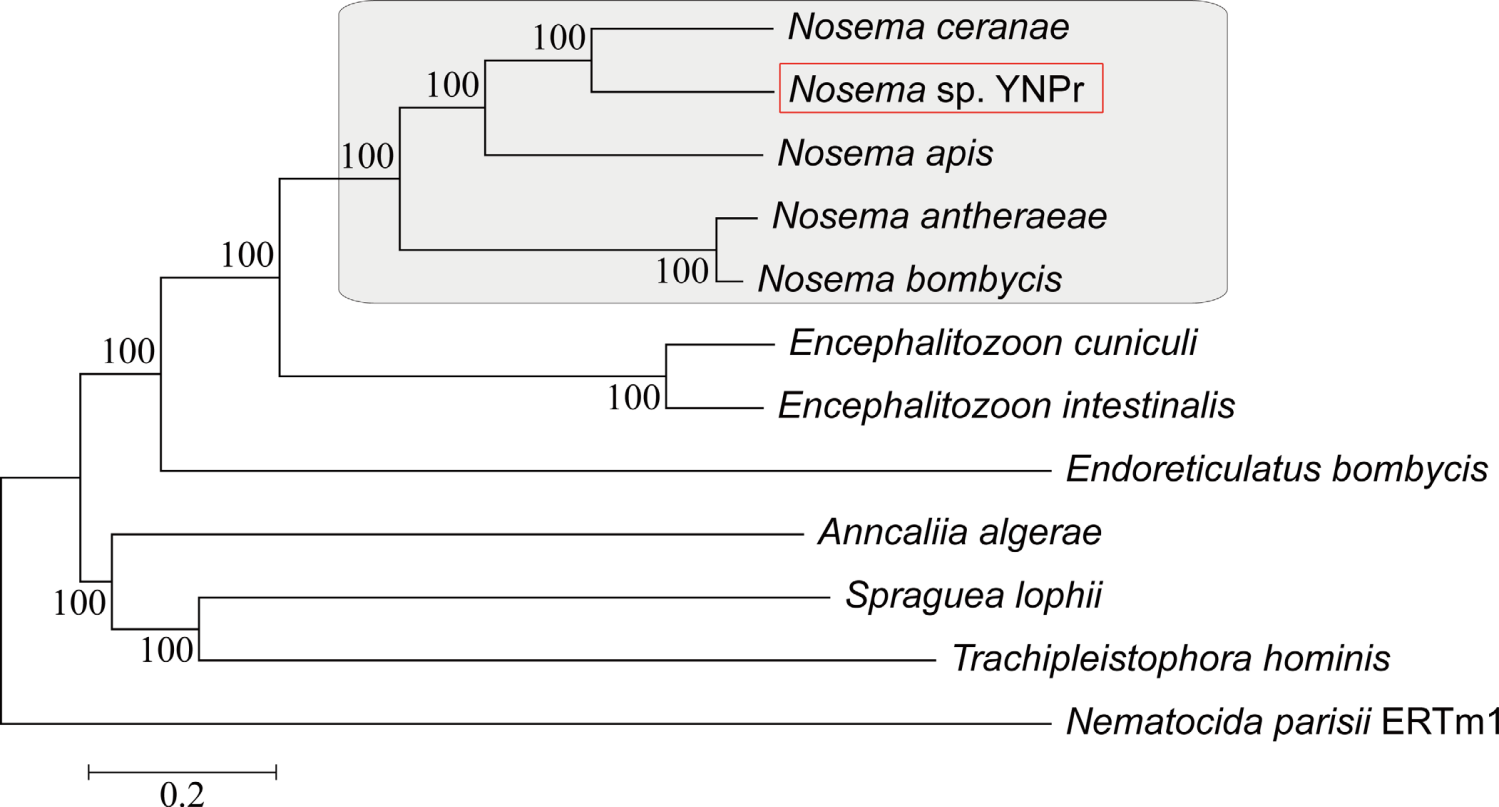
The evolution of microsporidia at the genome level. The maximum likelihood phylogeny of 12 microsporidia species based on a concatenation of common orthologous genes.

Neighbor Joining analysis using homologous genes shows a closer relationship between *N*. *ceranae* and *Nosema* sp. YNPr than between *N*. *ceranae* and *N*. *apis* (both honeybee parasites) and a close relationship between the two silkworm parasites *N*. *antheraeae* and *N*. *bombycis* (Fig **2**). The same phylogenetic relationships were obtained using the SSU rDNA and LSU rDNA genes only (S1A and S1B Fig). The arrangement of the functional ribosomal RNA operon from *Nosema* sp. YNPr (5′-SSU-ITS-LSU-3′) is the same as that found in most members of the *Nosema/Vairimorpha* clade [58,59,60]. The ITS sequence of *Nosema* sp.YNPr from Yunnan Province is identical to that of a previously reported isolate, *Nosema* sp. MPr from the same host species (*P*. *rapae*) but from Jiangsu province (S1C Fig)[61]. It is clear, as shown in S1A Fig, that genome size changes quite rapidly even between closely related species.

### Genomic architecture of *Nosema* sp. YNPr

Sequencing and assembly statistics are summarized in Table 1. The random genomic *Nosema* sp. YNPr library of 800 bp inserts yielded 3,154,248 reads and was assembled into 462 scaffolds. The combined scaffold length was 3.36 Mb. The transposable element (TE) content of the assembly was used to approximate the total TE content of the genome and the inferred genome size is contingent on the accuracy of this assumption. The N50 of the *Nosema* sp. YNPr genome is 12,222 bp; in comparison the N50 of *N*. *apis* is 24,309 bp and that of *N*. *ceranae* is 2,902 bp. The longest scaffold size is 45,514 bp and the mean scaffold length is 7,874 bp. The mean sequence coverage of scaffolds was 90 X. The G+C content of the *Nosema* sp. YNPr genome is 23.19%, which is lower than those of *N*. *ceranae* (26%), *N*. *bombycis* (31%) and *N*. *antheraeae* (28%) but higher than that of *N*. *apis* (18.78%) [54,55,56]. The protein coding regions have a significantly higher G+C content (25.53%) than does the overall genome.

**Table 1:** Genome assembly statistics for *Nosema* sp. YNPr.

General characteristics	*Nosema* sp. YNPr
Clean reads	3,154,248
Assembled (Mb)	3.36
Genome coverage	90X
G+C content (%)	23.19
Total number of scaffold	462
Length of scaffold (bp)	654~45,514
Scaffold N50 (bp)	12,222
No.of CDS	2,075
Mean CDS length (bp)	969
Mean size of scaffold (bp)	7,874

We identified 2075 ORFs in the *Nosema* sp. YNPr genome, with a mean length of 969 bp (Table 1). Based on our analysis there are 1,425 ORFs containing the CCC or GGG motifs in the genome. and An additional 275 CDS are predicted based on an an AT content >80% in the region 50 nucleotides upstream of an AUG codon within the ORF. (S1 Table, S2 Fig). All of the genes involved in core carbon metabolic pathways previously published from microsporidial genomes have been identified in *Nosema* sp. YNPr. The inability to identify some of the hypothetical genes using BLAST can be explained either by the highly divergent nature of the microsporidia resulting in low similarity with known genes or as genes specific to the microsporidia.

**Fig 3:**
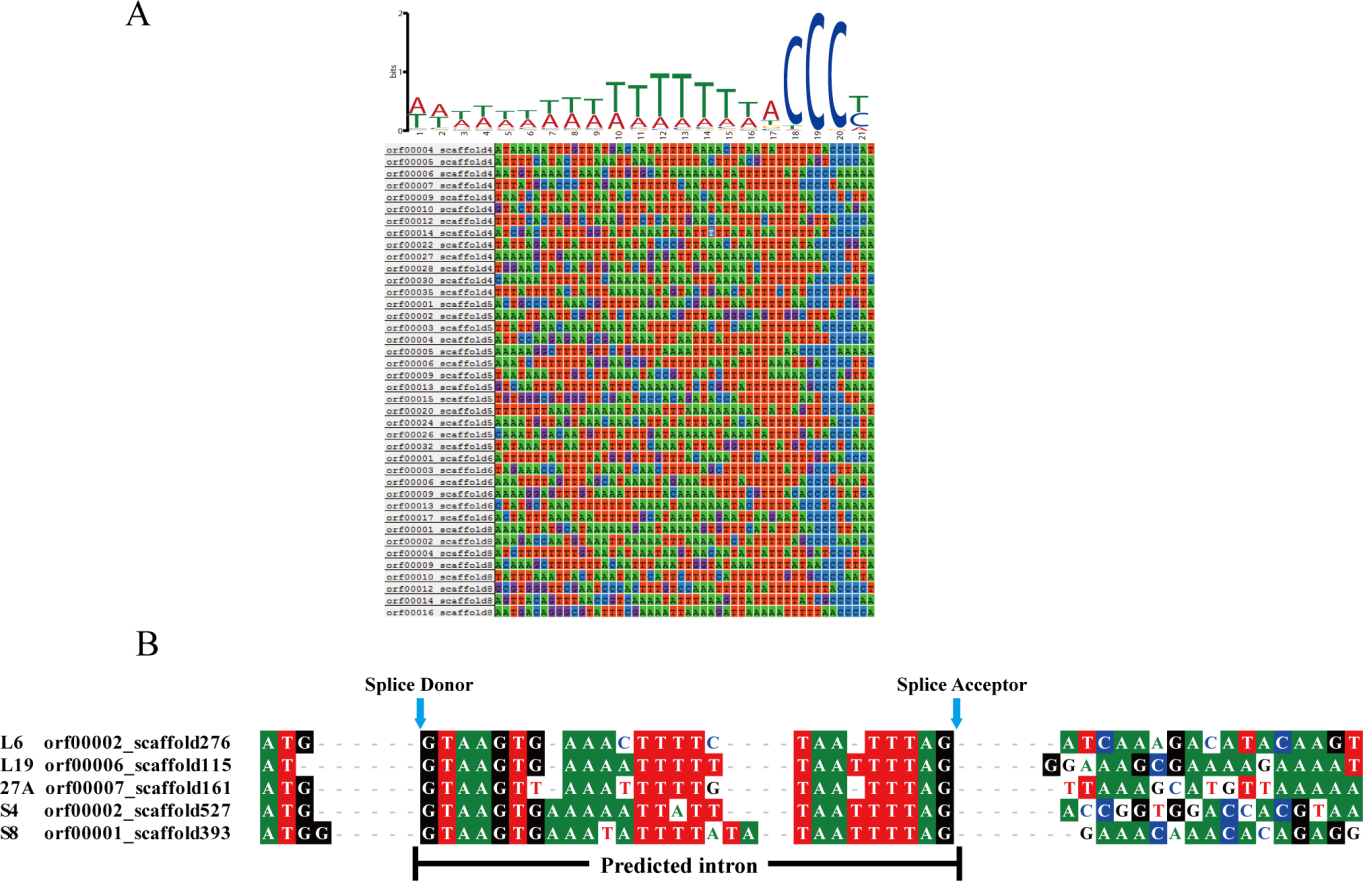
Noncoding regions of selected *Nosema* sp. YNPr genes. A. Putative regulatory C-rich motif in the region upstream of the start codon in *Nosema* sp. YNPr. B. Comparison of the regions flanking the introns for five *Nosema* sp. YNPr genes.

Results of the analysis for the ACCCTT motif approximately 50 bp upstream of the start codon in *Nosema* sp. YNPr is shown in Fig 3A. This motif is conserved in *N*. *ceranae*, *N*. *apis* and *E*. *cuniculi* and was present in 60% of the predicted genes for *Nosema* sp. YNPr [62,63,35]. A search for homology with the genes of *N*. *ceranae* identified five intron-containing ribosomal protein genes in *Nosema* sp. YNPr. They all contain **GT**AAGT at the donor site and TT**AG** at the acceptor site (Fig 3B). Two of the proteins (*L19*, *S4*) have orthologous intron-containing genes in *N*. *apis* and *N*. *bombycis* while homologues of the other three proteins (*L6*, *27A*, *S8*) do not contain introns in *N*. *apis* and *N*.*bombycis*.

### Comparison of functional genes

**Fig 4:**
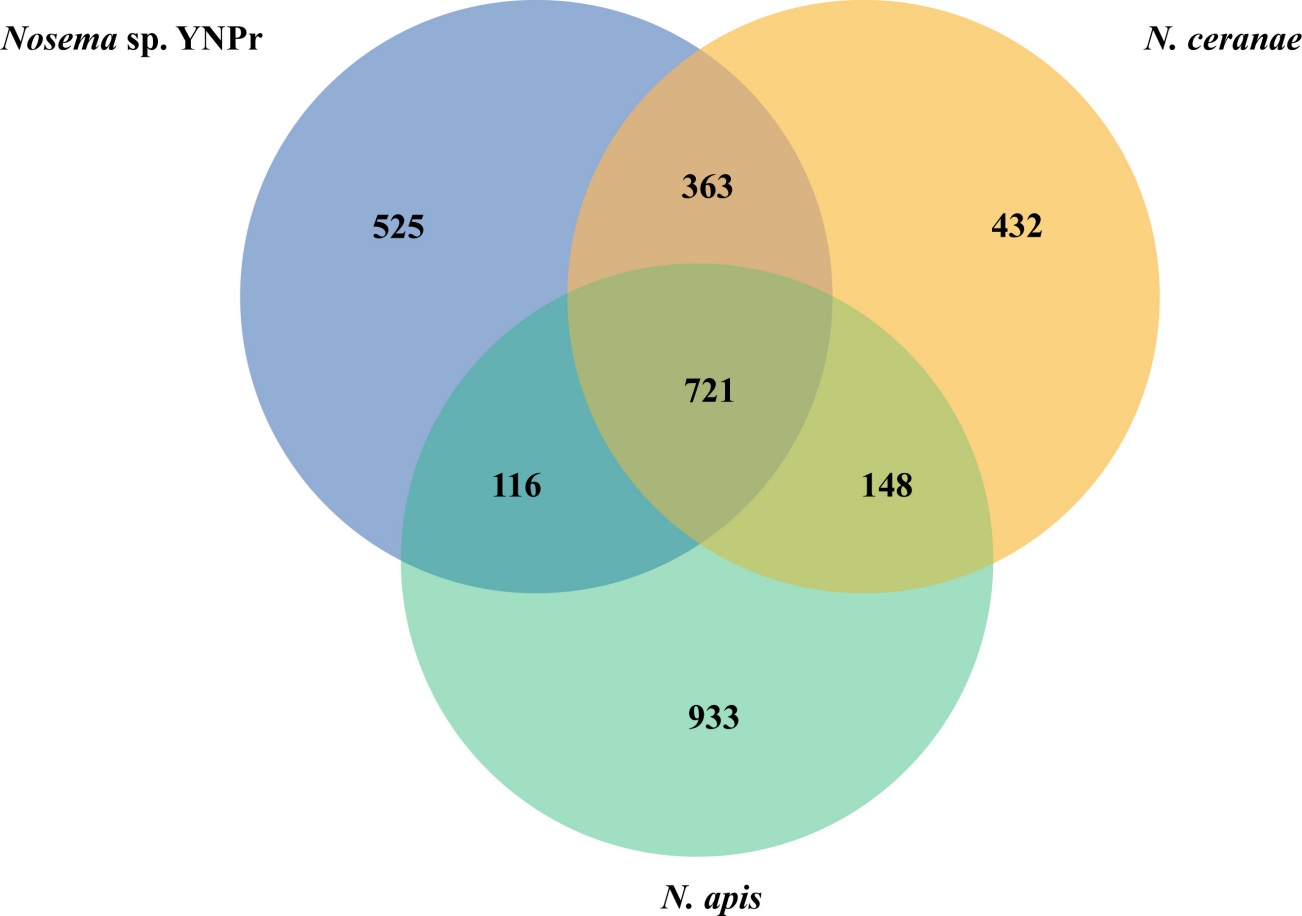
Venn diagram comparing the genes of three species of microsporidia. The number of homologous genes and species-specific genes among *Nosema* sp. YNPr, *Nosema ceranae* and *Nosema apis* are shown.

**Fig 5:**
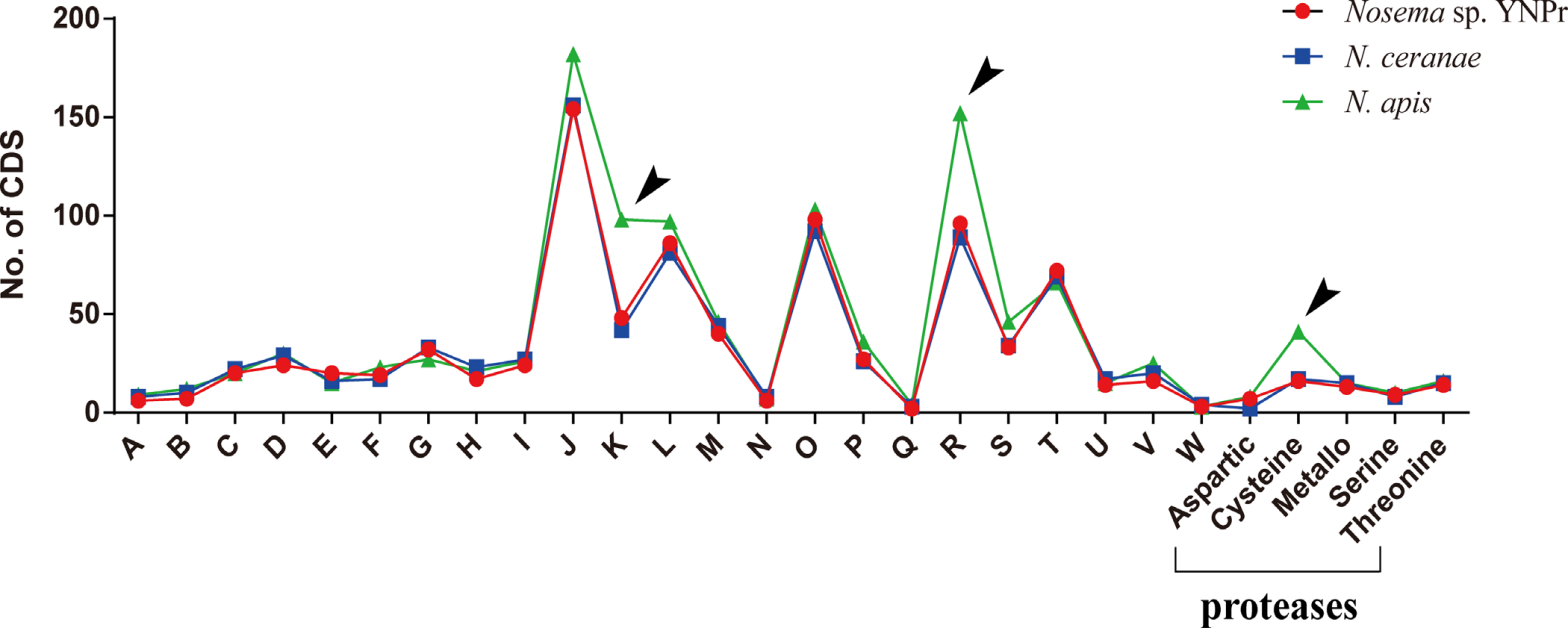
Comparison of coding sequence (CDS) numbers based on COG annotation and protease class among *Nosema* sp. YNPr, *N*. *ceranae* and *N*. *apis*. Black arrows indicate gene function categories for which *Nosema sp*. YNPr and *N*. *ceranae* differ in number of coding sequences from *N*. *apis*. A: RNA processing and modification; B: Chromatin structure and dynamics; C: Energy production and conversion; D: Cell cycle control, cell division, chromosome partitioning; E: Amino acid transport and metabolism; F: Nucleotide transport and metabolism; G: Carbohydrate transport and metabolism; H: Coenzyme transport and metabolism; I: Lipid transport and metabolism; J: Translation, ribosomal structure and biogenesis; K: Transcription; L: Replication, recombination and repair; M: Cell wall/membrane/envelope biogenesis; N: Cell motility; O: Posttranslational modification, protein turnover, chaperones; P: Inorganic ion transport and metabolism; Q: Secondary metabolites biosynthesis, transport and catabolism; R: General function prediction only; S: Function unknown; T: Signal transduction mechanisms; U: Intracellular trafficking, secretion, and vesicular transport; V: Defense mechanisms; W: Extracellular structures.

Comparison of genes homologous among *Nosema* sp. YNPr, *N*. *ceranae* and *N*. *apis* revealed that there are 721 genes shared among all three species, 525 genes that are *Nosema* sp. YNPr-specific, 432 that are *N*. *ceranae* -specific, and 933 that are *N*. *apis*-specific (Fig 4). Results of the COG protein database analysis indicate that the various cellular functions are similar in almost all categories except for Transcription (K) and General function prediction (R) which are substantially more numerous in *N*. *apis* than in the other two species (Fig 5). Cysteine proteases are also more abundant in *N*. *apis* than in *N*. *ceranae* and *Nosema* sp. YNPr (Fig 5).

### Variation in Genome Composition

#### Decrease in gene length

**Fig 6:**
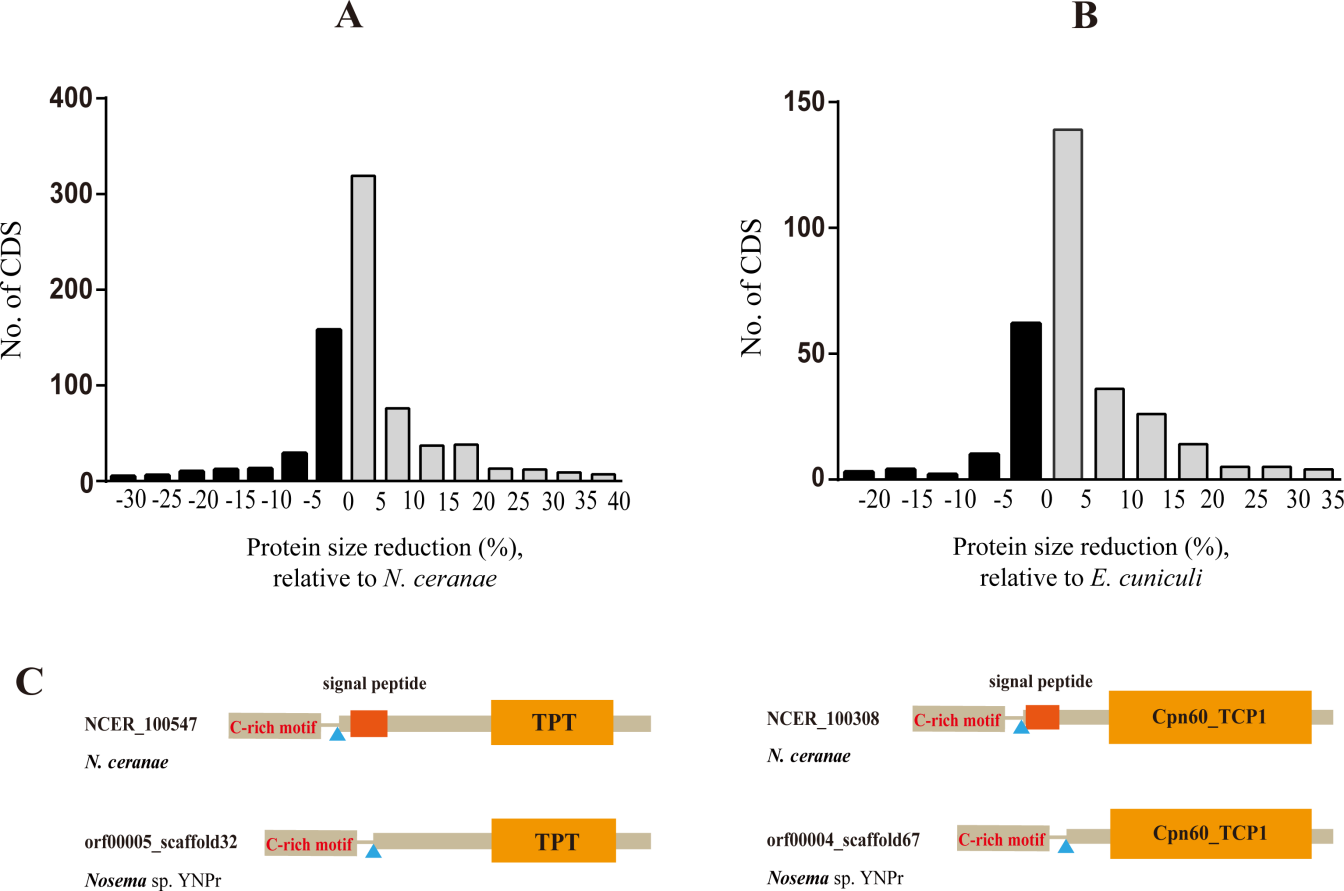
Comparison of CDS size between *Nosema* sp. YNPr and two other microsporidia. A. Degrees of reduction in length of *Nosema* sp. YNPr (Np) CDS relative to those of *N*. *ceranae* (Nc). X-axis value is expressed as percentage: 100 (Nc CDS length–Np CDS length)/(Nc CDS length). B. Degrees of reduction in length of *Nosema* sp. YNPr CDS relative to those of *E*.*cuniculi* (Ec). X-axis value is expressed as percentage: 100 (Ec CDS length–Np CDS length)/(Ec protein length). The positive classes representative of shorter *Nosema* sp.YNPr CDS are in grey. C. Two cases showing the signal peptide deletions in *Nosema* sp. YNPr relative to those of *N*. *ceranae*.

We compared the lengths of 1084 genes common to *Nosema* sp. YNPr and *N*. *ceranae* and found that 75% of the genes were shorter in *Nosema* sp. YNPr (Fig 6A). The total coding region of the genome was 364,065 amino acids in *Nosema* sp. YNPr and 368,901 amino acids in *N*. *ceranae*. Similarly, when 340 genes common to *Nosema* sp. YNPr and *E*. *cuniculi*, the closest sister taxon to the *Nosema/Vairimorpha* clade, were compared, it was found that 74% of the genes were smaller in *Nosema* sp. YNPr than in *E*. *cuniculi* (Fig 6B). The total coding region of the genome was 126,156 amino acids in *Nosema* sp. YNPr and 131,659 amino acids in *E*. *cuniculi*. An analysis of the common genes that are shorter in *Nosema* sp. YNPr than in *N*. *ceranae* shows a loss of signal sequences in *Nosema* sp. YNPr (Fig 6C) rather than a loss of individual amino acids.

#### Signal Peptides

The number of genes with signal peptides varies widely among species in the *Nosema/Vairimorpha* clade (S2 Table). Closely related species show similar numbers and percentages of genes containing signal peptides. *Nosema bombycis* and *Nosema antheraeae* have 431 and 394 genes with signal peptides respectively, while *Nosema* sp. YNPr and *Nosema ceranae* have109 and 159 respectively. S3 Table compares homologous genes of *Nosema* sp. YNPr and *Nosema ceranae* that contain predicted signal peptides in one or both species. Twenty-three genes in *Nosema* sp. YNPr lack the signal peptide seen in *Nosema ceranae*, while 5 genes in *Nosema ceranae* lack the signal peptide seen in *Nosema* sp. YNPr.

#### Decrease in size of intergenic regions

**Fig 7:**
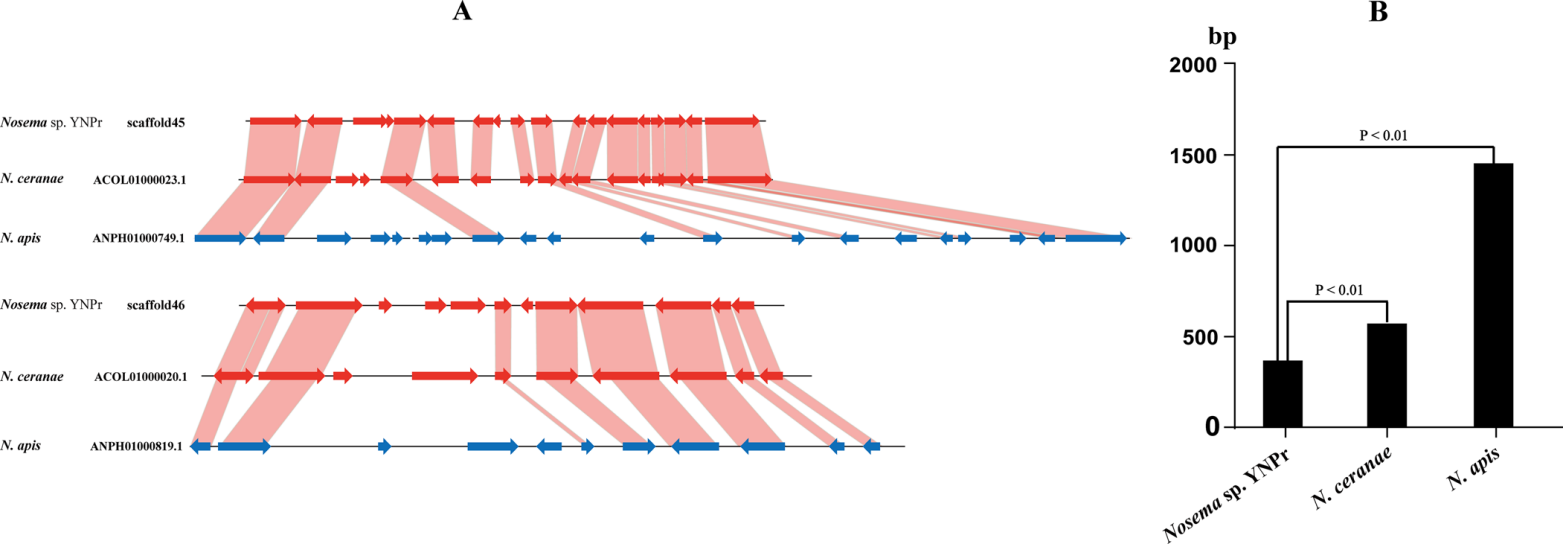
Syntenic, intergenic and overall size comparisons of *Nosema* sp. YNPr scaffolds with those of *N*. *ceranae* and *N*. *apis*. A. Syntenic and intergenic comparisons of two *Nosema* sp. YNPr scaffolds with those of *N*. *ceranae* and *N*. *apis*. B. Comparison of average intergenic lengths in *Nosema* sp. YNPr, *N*. *ceranae* and *N*. *apis*.

Syntenic comparisons among *Nosema* sp. YNPr, *N*. *ceranae* and *N*. *apis* indicate that genes are more tightly arranged (compacted) in *Nosema* sp. YNPr and *N*. *ceranae* than in *N*. *apis* (Fig 7A). The average length of the intergenic regions for the two scaffolds shown is significantly greater in *N*. *apis* (1438 nucleotides) than in *Nosema* sp. YNPr (357 nucleotides) and *N*. *ceranae* (607 nucleotides) (Fig 7B).

#### Transposon numbers

A search of the assembled *Nosema* sp. YNPr genome for repetitive DNA yielded a number of transposon types including LTR, Merlin, Tc1/mariner, LINE and Helitron (Table 2). The transposon content is 4.8% in *Nosema* sp. YNPr, 3.7% in *N*. *ceranae*, and 2.5% in *N*.*apis*. *Nosema* sp. YNPr and *N*. *ceranae* possess all 5 of the above mentioned transposon types but the Merlin, Tc1/mariner and Helitron classes are missing from *N*. *apis*. For the transposon types shared among these species there is a high variability in copy number. Analysis of the reverse transcriptase of the long terminal repeat (LTR) transposons shows that they cluster into 4 major groups (S3 Fig). The LTR copy number in *N*. *bombycis*, a sister taxon to the three *Nosema* species analyzed here, is much higher indicating that LTR transposons account for some of the differences in genome size in closely related taxa. Of note also is the absence of Group II LTR sequences in *N*. *apis*.

**Table 2:** Transposon types in three *Nosema* species.

Type	*Nosema* sp. YNPr	*N*. *ceranae*	*N*. *apis*
LTR	107103	99104	52531
Merlin	829	1165	0
Tc1/mariner	1983	36357	0
LINE	9756	27203	13496
Helitron	1190	453	0
Others	42596	126319	143533
Total	163457(4.8%)	290601(3.7%)	209560(2.5%)

## Discussion

Microsporidia have highly reduced genomes [36,39]. These obligate intracellular parasites can use host metabolites for their own cellular processes [41]. Genome compaction in the microsporidia has been studied extensively in terms of biochemical pathways and minimal genome sizes [37,39]. These studies show that the microsporidia have a core set of genes and an expanded set of cell surface transporters which allow them to import metabolic precursors from the host instead of producing these molecules themselves. The highly dynamic nature of genome evolution in the microsporidia is discussed in terms of gene compaction, size of intergenic regions, introns and overlapping genes [39].

Microsporidial genomes, though small, change in a dynamic fashion and have been shown in some cases to expand and in others to contract over time (S1 Fig) [39]. The fact that closely related microsporidial species have a number of unique genes (Fig 4) suggests that genes may be gained and lost during the process of host switching. Studies are needed to elucidate the functions of these species-specific genes

Our results show that *Nosema* sp. YNPr has shorter genes (Fig 6), shorter intergenic regions (Fig 7) and fewer transposons than do *N*. *apis* and *N*. *ceranae*. Gene size appears to be decreasing through loss of domains, including signal peptides (Fig 6). In a comparison of homologous genes between *Nosema* sp. YNPr and *N*. *ceranae*, 23 of the *Nosema* sp. YNPr genes lacked the signal peptide present in the *Nosema ceranae* homologue(S3 Table), indicating that the signal peptide content of homologous genes in closely related species can change rapidly. However, the total number of genes (both homologous and unique) containing signal peptides is similar in closely related species (S2 Table).

Phylogenetic analysis shows *Nosema* sp. YNPr and *N*.*ceranae* to be more closely related than are the two honeybee parasites, *N*. *ceranae* and *N*. *apis*. This relationship provides evidence of host switching across the insect orders Lepidoptera and Hymenoptera in the *Nosema/Vairimorpha* clade. A search of the protease database shows that *N*. *apis* has substantially more cysteine proteases than do either *N*. *ceranae* or *Nosema* sp. YNPr (Fig 5). Cysteine proteases are among the main proteolytic enzymes found in many protozoan parasites, and cysteine protease inhibitors have been shown to be effective against a variety of protozoans including *Trypanosoma cruzi* [64], *Entamoeba histolytica* [65] and *Plasmodium falciparum* [66].

The transposon makeup of these *Nosema* species varies widely and would seem likely to play a large role in genome evolution. *N*. *apis* appears to have no Merlin, Tc1/mariner or Helitron transposons, all of which are present in both *Nosema* sp. YNPr and *N*. *ceranae* (Table 2). These differences in transposon content among closely related species indicate that transposons can move in and out of genomes rapidly over relatively short evolutionary time periods. Determining the roles of these transposons in the adaptation of a microsporidial parasite to its host and in genome expansion and contraction in the microsporidia would be illuminating.

## Conclusion

Microsporidia comprise over 1,300 species and infect hosts from every animal phylum from marine, freshwater and terrestrial habitats. Because of their rich host diversity they are an excellent model system for the study of interactions between obligate single-celled parasites and their hosts at many levels. The *Nosema/Vairimorpha* clade encompasses a wide-ranging group of parasites from a diverse collection of hosts in which host-parasite co-evolutionary principles can be tested. Members of the *Nosema/Vairimorpha* clade have been reported from Lepidoptera, Coleoptera, Hymenoptera and other invertebrates including mites [27,67,68]. From our phylogenetic analyses and those of others [39,69,70] it appears that host switching occurs relatively rapidly over evolutionary time in the microsporidia. We hypothesize that natural selection plays a role in the evolution of the small yet dynamically changing microsporidial genomes and suggest that there may be a trade-off between smaller genomes for rapid reproduction and larger genomes with more genetic options with which to challenge a host. However, in order to examine the role of genetic drift versus natural selection in microsporidial genome evolution it will be necessary to sequence and analyze additional genomes from the *Nosema/Vairimorpha* clade. Analyses would include determining dN/dS ratios, functions of genes unique to closely related species [39] in different hosts, genome size versus host persistence, and the searching for convergence in microsporidia from different genera that infect the same host.

## Supporting Information

S1 Fig Phylogenetic analysis for rDNA sequence. A: Phylogenetic tree of SSU rDNA; B: Phylogenetic tree of LSU rDNA tree; C: Multiple sequence alignments of ITS between *Nosema* sp. YNPr and *Nosema* sp. MPr.
(TIF)

S2 Fig GO annotation of *Nosema* sp. YNPr, *N*. *ceranae* and *Nosema apis* genomes. (TIF)

S3 Fig The cluster analysis of LTR conservative reverse transcriptase sequences. (TIF)

S1 Table The annotation of all genes in the *Nosema* sp. YNPr genome. (XLS)

S2 Table Numbers of genes containing signal peptides in *E*. *cuniculi* and five members of the *Nosema/Vairimorpha* clade. (DOC)

S3 Table Prediction of Signal Peptides in homologues from *Nosema* sp.YNPr and *Nosema ceranae*. (DOC)
