# Supplementary figures and images for: The Genome of Nosema sp. Isolate YNPr: A Comparative Analysis of Genome Evolution within the Nosema/Vairimorpha Clade
Source: PLoS One. 2016 Sep 6;11(9):e0162336. doi: 10.1371/journal.pone.0162336 (PMC5012567; doi:10.1371/journal.pone.0162336)

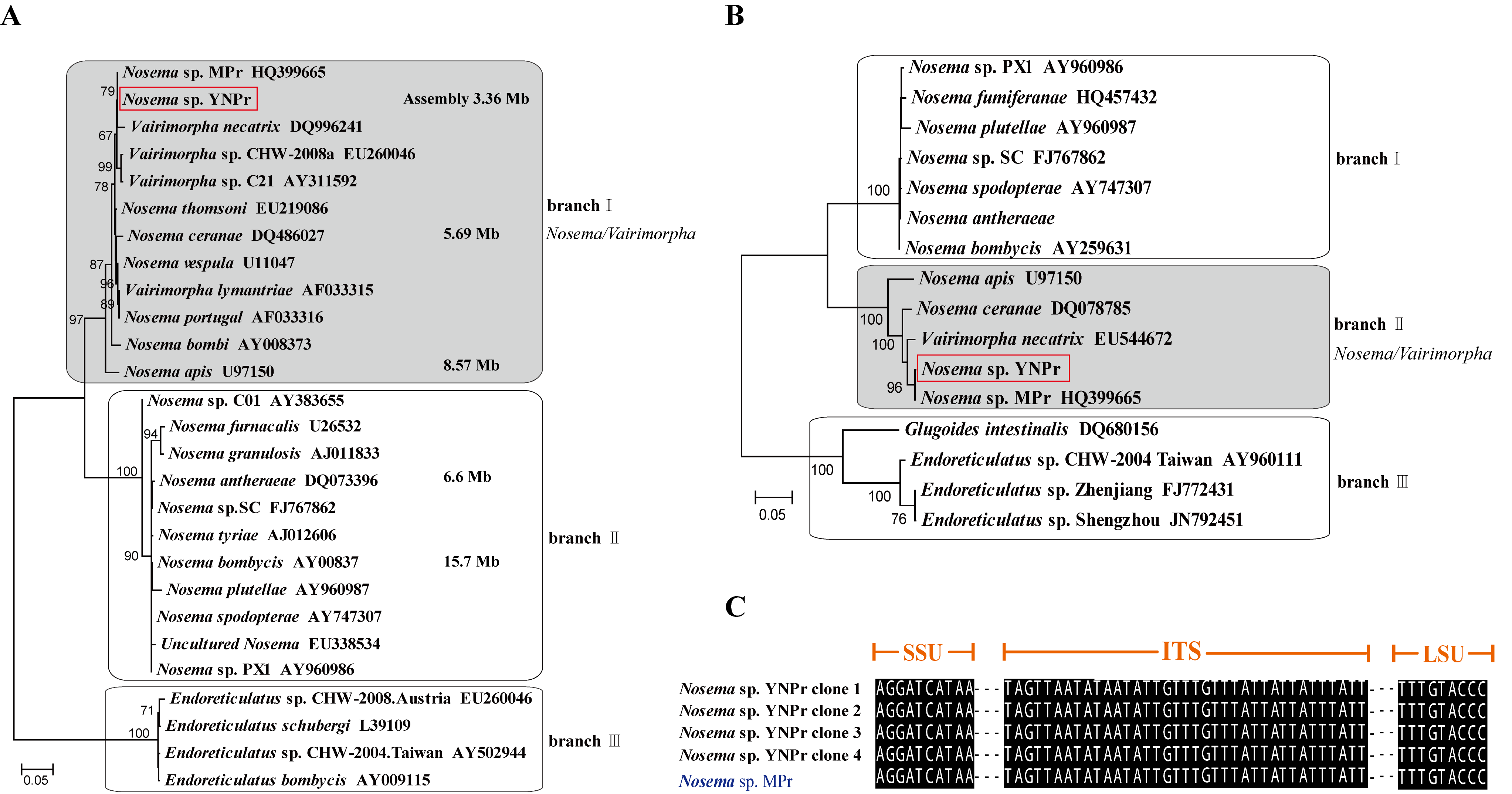

Supplement: S1 Fig — A: Phylogenetic tree of SSU rDNA; B: Phylogenetic tree of LSU rDNA tree; C: Multiple sequence alignments of ITS between Nosema sp. YNPr and Nosema sp. MPr. (TIF) [file pone.0162336.s001.tif]

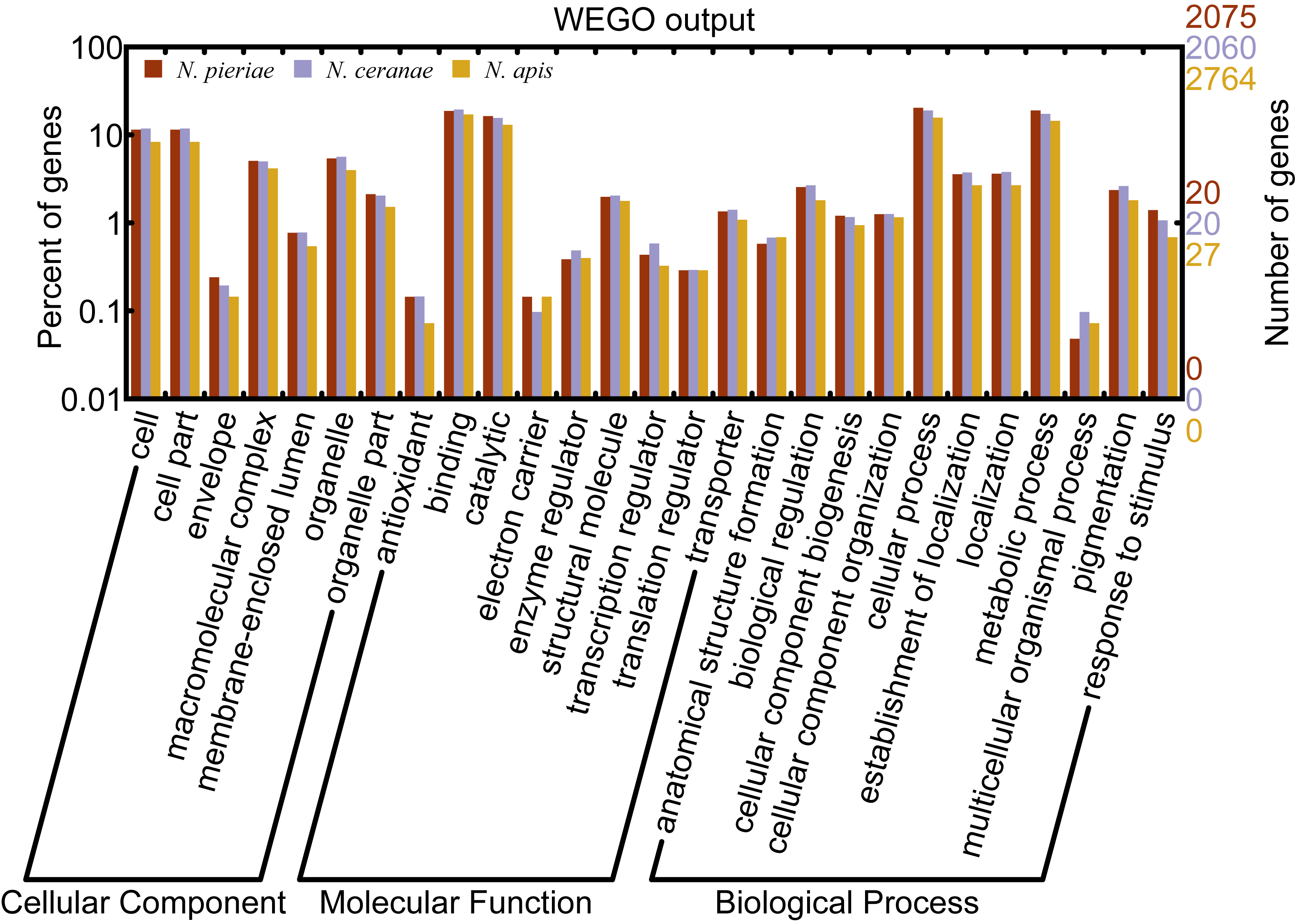

Supplement: S2 Fig — (TIF) [file pone.0162336.s002.tif]

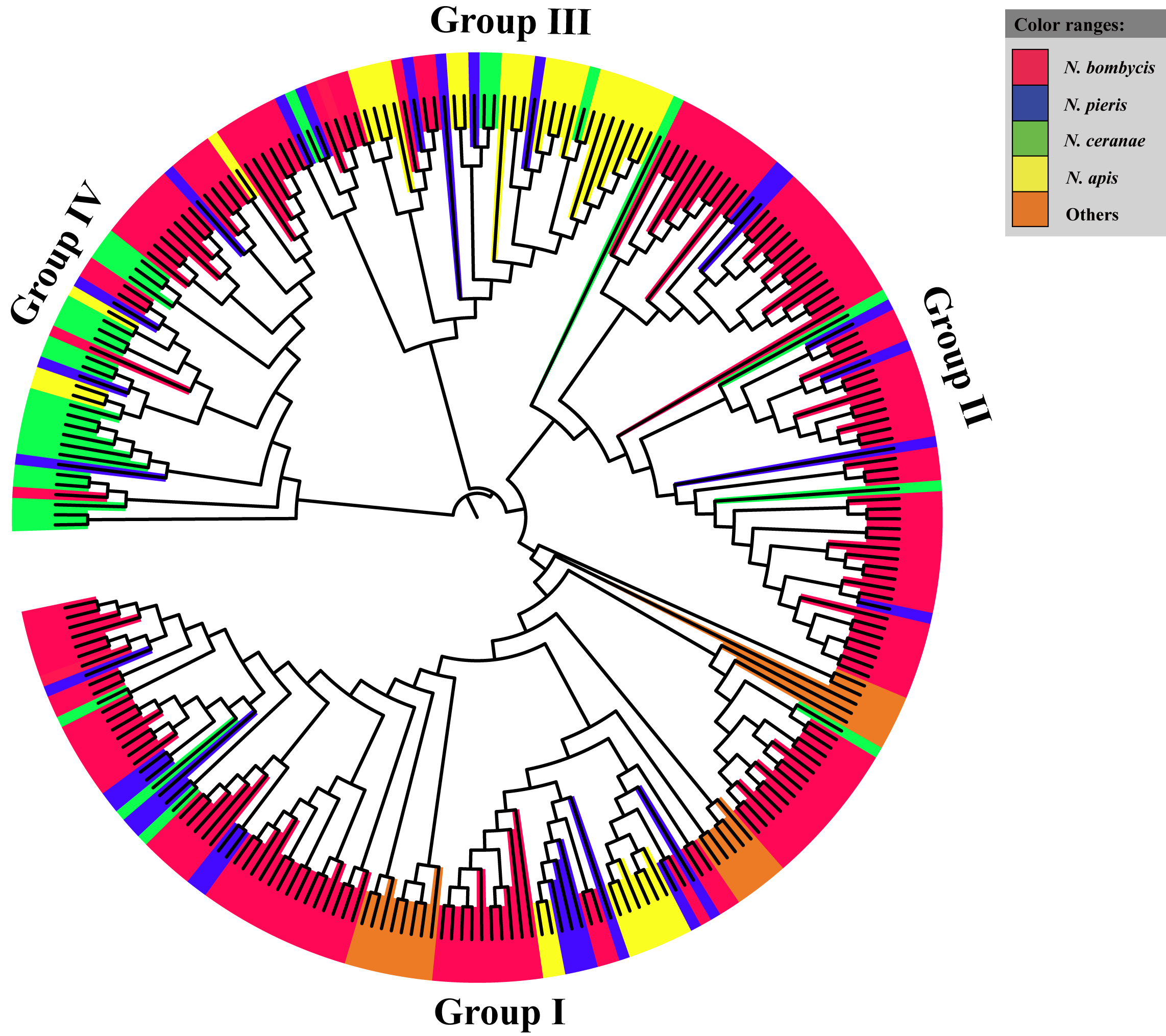

Supplement: S3 Fig — (TIF) [file pone.0162336.s003.tif]
