# Supplementary material for: The Genome of Nosema sp. Isolate YNPr: A Comparative Analysis of Genome Evolution within the Nosema/Vairimorpha Clade
Source: PLoS One. 2016 Sep 6;11(9):e0162336. doi: 10.1371/journal.pone.0162336 (PMC5012567; doi:10.1371/journal.pone.0162336)
Supplement: S2 Table — (DOC) [file pone.0162336.s005.doc]

| Species | Number and Percentage of genes (both homologous and unique) containing signal peptides |
| --- | --- |
| *Nosema* sp. YNPr | 109(5.3%) |
| *Nosema ceranae* | 159(7.7%) |
| *Nosema bombycis* | 431(9.7%) |
| *Nosema anthereaeae* | 394(10.2%) |
| *Nosema apis* | 132(4.8%) |
| *Encephalitozoon cuniculi* | 103(5.2%) |

**S2 Table. Numbers of genes containing signal peptides in *E. cuniculi* and 5 members of the *Nosema/Vairimorpha* clade.**
