# Supplementary material for: The Genome of Nosema sp. Isolate YNPr: A Comparative Analysis of Genome Evolution within the Nosema/Vairimorpha Clade
Source: PLoS One. 2016 Sep 6;11(9):e0162336. doi: 10.1371/journal.pone.0162336 (PMC5012567; doi:10.1371/journal.pone.0162336)
Supplement: S3 Table — (DOC) [file pone.0162336.s006.doc]

**S3 Table . Prediction of Signal Peptides in homologues from *Nosema* YNPr and *Nosema* *ceranae*.**

| Gene Function | Signal peptide | |
| --- | --- | --- |
|  | *Nosema* sp. YNPr | *N. ceranae* |
| Polysaccharide deacetylase domain-containing protein | - | *+* |
| Unknown | + | *+`* |
| Unknown | + | *+* |
| Uncharacterized protein ECU09_1880 | - | *+* |
| Spore wall protein 7 | + | *+* |
| Solute carrier family 35 member B1 | - | *+* |
| Heat shock protein | + | *+* |
| Probable proteasome subunit beta type-1 | - | *+* |
| 3-ketodihydrosphingosine reductase TSC10 | - | *+* |
| Serine/threonine-protein kinase/endoribonuclease IRE1 | - | *+* |
| Unknown | - | *+* |
| T-complex protein 1 subunit eta | - | *+* |
| Unknown | - | *+* |
| Unknown | + | *-* |
| T-complex protein 1 subunit epsilon | - | *+* |
| Dolichyl-phosphate-mannose--protein mannosyltransferase 2 | + | *+* |
| GPI-anchor transamidase | + | *+* |
| E3 ubiquitin-protein ligase RFWD3 | - | *+* |
| Unknown | - | *+* |
| Unknown | - | *+* |
| Unknown | - | *+* |
| Unknown | + | *-* |
| Putative subtilisin-like proteinase 2 | + | *-* |
| Peptidyl-prolyl cis-trans isomerase B | + | *+* |
| Putative subtilisin-like proteinase 1 | + | *-* |
| Unknown | - | *+* |
| Unknown | - | *+* |
| Protein sel-1 homolog 1 | + | *+* |
| N-acetylglucosaminyl-phosphatidylinositol de-N-acetylase | + | *+* |
| Unknown | + | *-* |
| Unknown | - | *+* |
| Coatomer subunit delta | - | *+* |
| Spore wall protein ECU02_0150 | - | *+* |
| Unknown | - | *+* |
| Unknown | + | *+* |
| Unknown | + | *+* |
| Unknown | - | *+* |
| Proteasome subunit alpha type-7 | - | *+* |
| Unknown | - | *+* |
